# Supplementary material for: Attitudes, Risk Factors, and Behaviours of Gambling among Adolescents and Young People: A Literature Review and Gap Analysis
Source: Int J Environ Res Public Health. 2021 Jan 22;18(3):984. doi: 10.3390/ijerph18030984 (PMC7908209; doi:10.3390/ijerph18030984)
Supplement: Supplementary file 1 [file ijerph-18-00984-s001.pdf]

Supplementary File 1

Table 1: Quality Ratings for Peer-reviewed Literature (Qualitative studies)

Rating Criteria<br>Author	Kim et al. (2017)	Kristianse et al. (2017)	Kristianse et al. (2018)	Nekich & Ohtshka (2016)	Rash & McGrath (2017)	Tagoe et al. (2018)	Wardle et al. (2019) *(Review)*									
1. Is the qualitative approach appropriate to answer the research question?	Y	Y	Y	Y	Y	Y	Y									
2. Are the qualitative data collection methods adequate to address the research question?	Y	Y	Y	Y	Y	Y	Y									
3. Are the findings adequately derived from the data?	Y	Y	Y	Y	Y	Y	Y									
4. Is the interpretation of results sufficiently substantiated by data?	Y	Y	Y	Y	Y	Y	Y									
5. Is there coherence between qualitative data sources, collection, analysis and interpretation?	Y	Y	Y	Y	Y	Y	Y									

Table 2: Quality Ratings for Peer-reviewed (Quant RCTs)

Rating Criteria / Author	Calado et al. (2020)	O' Loughin & Blaszczynski	Sirolo et al. (2019)															
1. Is randomization appropriately performed?	Y	Y	?															
2. Are the groups comparable at baseline?	Y	Y	Y															
3. Are there complete outcome data?	Y	Y	Y															
4. Are outcome assessors blinded to the intervention provided?	?	Y	?															
5. Did the participants adhere to the assigned intervention?	Y	Y	Y															

Table 3: Quality Ratings for Peer-reviewed Literature – (Quant Non-Randomised)

Rating Criteria<br>Author	Ciccarelli et al. (2019)	Dussault et al. (2019)	Li et al. (2018)	Pallesen et al. (2016)	Wu et al. (2019)											
1. Are the participants representative of the target population?	Y	Y	Y	Y	Y											
2. Are measurements appropriate regarding both the outcome and intervention (or exposure)?	Y	Y	Y	Y	Y											
3. Are there complete outcome data?	Y	Y	Y	Y	Y											
4. Are the confounders accounted for in the design and analysis?	Y	Y	Y	Y	Y											
5. During the study period, is the intervention administered (or exposure occurred) as intended?	Y	Y	Y	Y	Y											

Table 4: Quality Ratings for Peer-reviewed Literature (Quant Descriptive)

Rating Criteria / Author	Abdi et al. (2015)	Aguocha et al. (2019)	Aguocha et al. (2019)	Andrie et al. (2018)	Bozzato et al. (2020)	Buja et al. (2019)	Canale et al. (2016a)	Canale et al. (2016b)	Cosenza et al. (2019)	Deans et al. (2017)	DeCamp (2020)	Dixon et al. (2016)	Donati et al. (2020)	Dussault et al. (2019)	Elton-Marshall et al. (2016)	Estevez et al. (2019)
1. Is the sampling strategy relevant to address the research question?	Y	Y	Y	Y	Y	Y	Y	Y	Y	Y	Y	Y	Y	Y	Y	Y
2. Is the sample representative of the target population?	Y	Y	Y	Y	Y	Y	Y	Y	Y	Y	Y	Y	Y	Y	Y	Y
3. Are the measurements appropriate?	Y	Y	Y	Y	Y	Y	Y	Y	Y	Y	Y	Y	Y	Y	Y	Y
4. Is the risk of nonresponse bias low?	Y	Y	Y	Y	Y	Y	Y	Y	Y	Y	Y	Y	Y	Y	Y	Y
5. Is the statistical analysis appropriate to answer the research question?	Y	Y	Y	Y	Y	Y	Y	Y	Y	Y	Y	Y	Y	Y	Y	Y

Table 4: Quality Ratings for Peer-reviewed Literature (Quant Descriptive) cont.

Rating Criteria / Author	Estevez et al. (2020a)	Estevez (2020b)	Farhat (2020a)	Farhat (2020b)	Foster et al. (2016)	Gavriel-Fried et al. (2015)	Ginley et al. (2015)	Ginley et al. (2018)	Gonzalez-Roz et al. (2017)	Gonzalez-Sicilia (2020)	Gori et al. (2015)	Hanss et al. (2015)	Ho (2017)	Huic et al. (2017)	Jasoorya et al. (2017)	Jauregui (2019)
1. Is the sampling strategy relevant to address the research question?	Y	Y	Y	Y	Y	Y	Y	Y	Y	Y	Y	Y	Y	Y	Y	Y
2. Is the sample representative of the target population?	Y	Y	Y	Y	Y	Y	Y	Y	Y	Y	Y	Y	Y	Y	Y	Y
3. Are the measurements appropriate?	Y	Y	Y	Y	Y	Y	Y	Y	Y	Y	Y	Y	Y	Y	Y	Y
4. Is the risk of nonresponse bias low?	Y	Y	Y	Y	Y	?	Y	Y	Y	Y	Y	Y	Y	Y	Y	Y
5. Is the statistical analysis appropriate to answer the research question?	Y	Y	Y	Y	Y	Y	Y	Y	Y	Y	Y	Y	Y	Y	Y	Y

Table 4: Quality Ratings for Peer-reviewed Literature (Quant Descriptive) cont.

Rating Criteria / Author	Jauregui (2020)	Kalte-negger (2019)	Kam et al. (2017)	Kang (2019)	Kang (2020)	King & Delfabbro (2016)	Livazovich (2019)	Marchica et al. (2017)	Martinez-Loredo (2019)	McBride et al. (2016)	Melendez-Torres (2020)	Oksanene (2019)	Paleologou (2 019)	Passanisi (2019)	Pisarska & Ostaszewski (2020)	Raisamo (2020)	Rasanen (2016)
1. Is the sampling strategy relevant to address the research question?	Y	Y	Y	Y	Y	Y	Y	Y	Y	Y	Y	Y	Y	Y	Y	Y	Y
2. Is the sample representative of the target population?	Y	Y	Y	Y	Y	Y	Y	Y	Y	Y	Y	Y	Y	Y	Y	Y	Y
3. Are the measurements appropriate?	Y	Y	Y	Y	Y	Y	Y	Y	Y	Y	Y	Y	Y	Y	Y	Y	Y
4. Is the risk of nonresponse bias low?	Y	Y	Y	Y	Y	Y	Y	Y	Y	Y	Y	Y	Y	Y	Y	N	Y
5. Is the statistical analysis appropriate to answer the research question?	Y	Y	Y	Y	Y	Y	Y	Y	Y	Y	Y	Y	Y	Y	Y	Y	Y

Table 4: Quality Ratings for Peer-reviewed Literature (Quant Descriptive) cont.

Rating Criteria / Author	Reardon et al. (2019)	Rider et al. (2019)	Salolainen et al. (2018)	Sanscartier et al. (2019)	Sarti & Triventi (2017)	Sharman, Murphy et al. (2019)	Sharman, Butler & Roberts (2019) *(Review)*	Spangberg & Svensson (2019)	Vitaro et al. (2019)	Walters et al. (2019)	Weinberger et al (2015)	Yu & Fa (2019)	Yu & Ma (2019) *(Review)*	Zhai et al. (2019)	Zhai et al. (2020)		
1. Is the sampling strategy relevant to address the research question?	Y	Y	Y	Y	Y	Y	Y	Y	Y	Y	Y	Y	Y	Y	Y		
2. Is the sample representative of the target population?	Y	Y	Y	Y	N	Y	Y	Y	Y	Y	Y	Y	Y	Y	Y		
3. Are the measurements appropriate?	Y	Y	Y	Y	Y	Y	Y	Y	Y	Y	Y	Y	Y	Y	Y		
4. Is the risk of nonresponse bias low?	Y	Y	Y	Y	N	Y	Y	Y	Y	Y	?	Y	Y	Y	Y		
5. Is the statistical analysis appropriate to answer the research question?	Y	Y	Y	Y	Y	Y	Y	Y	Y	Y	Y	Y	Y	Y	Y		

Supplementary File 2

Table 1: Summary of included articles

Author(s) & year/ Study location	Article type	Aim	Key findings	Gaps identified by the authors
Abdi et al. (2015)<br><br>Ethiopia	Cross-sectional research	To examine the personal, social and environmental risk factors of problem gambling in four high schools in Ethiopia, among students	Males more at risk than females. Most common form of gambling cards followed by coin toss and pool. Personal feelings about gambling, social factors and environmental factors were all significant correlates of problem gambling.	**Knowledge gap [Intervention programs]**<br>Need for systematic design and evaluation of evidence-based educational interventions to prevent problem gambling among young people
Aguocha et al. (2019)<br><br>Nigeria	Cross-sectional research	To investigate the determinants of gambling among male secondary school students	Students over 18 and whose friend, parents, sibling gamble, were more likely to be currently engaged in gambling behaviour. Having friends that gambled, accepted gambling, and parents with a gambling problem, predicted gambling among students. The study concluded that gambling is common among students.	**Knowledge gap [Intervention programs]**<br>Need for systematic design and evaluation of evidence-based intervention programs for adolescents
Andrie et al. (2019)<br><br>Greece, Germany, Iceland, Netherlands, Poland, Romania and Spain	Cross-sectional research	To examine gambling involvement and problem gambling correlates among European adolescents	Males higher risk than females, older age, parental education lower, and absence of siblings I.e being an only child. Higher risk gambling was associated with problematic internet use, lower grades, lower age when first using the internet. Conclusion, better gambling legislation to protect adolescent gambling involvement especially males	**Knowledge gap [more research in general]**<br>A need for more gambling research in general in countries where higher gambling involvement is lagging behind gambling regulation.
Bozzato et al. (2020)<br><br>Italy	Cross-sectional research	To examine gambling behaviour in adolescents: prevalence and its relation to social self-regulatory and academic self-efficacy	Higher risk and gambling more than one form of gambling was more common among males. 77% reported gambling at least once during the past 12 months (87.2% males; 67.1% females). The researchers suggested this rate was higher than other studies as lotteries and cards with family and friends were included – all types of gambling gambles. Males gambled to win money, taste of challenge, and to imitate friends. Females motivation was boredom (regulate emotion) and because family members do it.	**Knowledge gap [specific traits]**<br>More research on relationship between self-efficacy and gambling among adolescents
Buja et al. (2019)	Cross-sectional research	To examine the pathways connecting socioeconomic	Gambling associated with alcohol consumption. Students with higher income more at risk of	**Knowledge gap [Intervention programs]**<br>Need for development and evaluation of evidence-based

Italy		variables, substance abuse and gambling behaviour among Italian high-school students	gambling, drinking and smoking. Males more likely to be involved in risky behaviours. Females more likely to smoke. Students with better-educated parents were higher risk of alcohol and cannabis.	intervention programs for at risk youth
Calado et al. (2020)<br><br>Portugal	Pre-post intervention study	To investigate the efficacy of a gambling prevention program which targeted misconceptions about gambling and other risk behaviours, improving decision making skills	The study provides evidence for the effectiveness of gambling education programs in high school curricula.	**Knowledge gap [intervention programs]**<br>Further research to explore the relationship adolescent gamblers have with significant others and how this affects their attitudes and perceptions. Also, further research to develop and evaluate intervention programs.
Canale, Vieno et al. (2016)<br><br>Italy	Cross-sectional research	To understand the impact of online gambling on gambling problems in a large-scale nationally representative sample of Italian youth, and to identify and then further examine a subgroup of online gamblers who reported higher rates of gambling problems	Rates of problem gambling were five times higher among online gamblers than non-online gamblers. Factors that increased the risk of becoming a problem online gambler included living with non-birth parents, having a higher perception of financial family status, being more involved with gambling, and the medium preferences of remote gamblers (e.g., Internet cafes, digital television, and video game console).	**Knowledge gap [Sub-populations]**<br>Future studies should quantify the extent of involvement in online gambling to enable evaluation of regular Internet gambling.<br>The comparisons between the more nuanced groups of gamblers (offline gambling only, online gambling only, online and offline gambling but on different activities, online and offline gambling but on the same activities) warrant additional study.<br>Future studies may benefit from including impulsivity traits and reasons for gambling to better elucidate the differences between online and non-online gamblers.
Canale et al. (2016)	Cross-sectional research	To understand the impact of online gambling on gambling problems in a large-scale nationally representative sample of Italian youth, and to identify and then further examine a subgroup of online gamblers who reported higher rates of gambling problems	Rates of problem gambling were five times higher among online gamblers than non-online gamblers. Factors that increased the risk of becoming a problem online gambler included living with non-birth parents, having a higher perception of financial family status, being more involved with gambling, and the medium preferences of remote gamblers (e.g., Internet cafes, digital television, and video game console).	**Knowledge gap [Sub-populations]**<br>Future studies should quantify the extent of involvement in online gambling to enable evaluation of regular Internet gambling.<br>The comparisons between the more nuanced groups of gamblers (offline gambling only, online gambling only, online and offline gambling but on different activities, online and offline gambling but on the same activities) warrant additional study.<br>Future studies may benefit from including impulsivity traits and reasons for gambling to better elucidate the differences between online and non-online gamblers.
Ciccarelli et al. (2019)<br><br>Italy	Non-randomised experimental study	To examine attentional bias among adolescent problem gamblers and non-gamblers	Problem gamblers displayed greater bias towards attending to gambling related cues than non-problem gamblers.	**Knowledge gap [attentional bias among adolescent gamblers]**<br>Further research on the precise nature of relationship between adolescent biases of attention re gambling stimuli
Cozensa et al. (2019)<br><br>Italy	Cross-sectional design	To investigate the role that general impairment in mentalization plays in gambling disorder	Results indicated that problem gamblers tended to focus more on the immediate outcomes of their behaviour or reward/outcome than non-problem gamblers.	**Knowledge gap [more research in general]**<br>More research on how adolescents mentalise and perceive gambling
Deans et al.	Qualitative	To explore the way peer	Respondents perceived that sports betting was a	**Knowledge gaps [Research preventative programs]**

(2017)<br><br>Australia	research	group behaviours influenced attitudes towards, and the consumption of, gambling products among young sports betting gamblers	normal and accepted activity, embedded within sport.	Need for development and evaluation of public health campaigns as a part of a public health approach to address the impact of marketing tactics in shifting social norms
DeCamp (2020)<br><br>USA	Cross-sectional research	To examine the association between gaming loot boxes and problem gambling	In contrast to other loot box studies that examined adults, a lower prevalence of loot box purchase was observed among youth gamers i.e. a lower proportion of youth gamers purchased loot boxes than adult gamers. Overall, loot boxes and gambling share similarities, however, risk and protective factors may not be the same.	**Knowledge gap [more research in general]**<br>Large knowledge gap in area of loot-boxes particularly given the prevalence of them among internet games
Derevensky et al. (2019)<br><br>Canada	Discussion article	Discussion of problem gambling gaming, internet, and smartphone use among children and adolescents	Changing face of gambling due to technological advances revolutionising the gambling industry. Very few adolescent children and adolescents voluntarily seek treatment for behavioural disorders. Parents play an essential role in helping prevent and modify their children's behavioural problems/addictions.	**Knowledge gap [longitudinal research]**<br>Further examination of the developmental trajectories and risk and protective factors for smartphone addiction and online gambling addiction needed
Derevensky et al. (2015)<br><br>Canada	Discussion paper	Discussion paper of adolescent gambling literature	More forms of gambling now available, and the social acceptability of gambling has resulted in more adolescents engaging in gambling, despite the 18 years old age restriction. The term 'gaming' helps to normalise gambling as a socially acceptable form of entertainment. Young people are reluctant to seek treatment for problem gambling.	**Knowledge gap [more research in general]**<br>More research needed given rapidly changing gambling environment
Dixon et al. (2016)<br><br>Australia	Cross-sectional research	Examination of the relationship between gambling attitudes, involvement, and problems in adolescence	Parental discipline and adolescent coping styles protected against at-risk gambling for those who gambled infrequently but did not protect those adolescents where gambling involvement was high.	**Knowledge gap [more research in general]**<br>More research on influence of parental attitudes and adolescent gambling involvement
Donait et al. (2020)<br><br>Italy	Cross-sectional research	To examine immigration status on sensation seeking and problem gambling	Immigrant adolescents had higher problem gambling and risky behaviours than non-immigrants. Moderation analyses showed higher sensation seeking related to problem gambling after controlling for gender for immigrants.	**Knowledge gap [Intervention programs]**<br>More research on sensation seeking and problem gambling among this target group – immigrants
Donait et al.	Cross-sectional	To examine time perspectives	Results showed that the present time perspective	**Knowledge gap [Intervention programs]**

(2019)<br><br>Italy	research	and gambling in adolescent boys	was associated with problem gambling, and future time perspective negatively associated.	Development and evaluation of intervention programs which help adolescents focus on future goals, to mitigate risk of development of problem gambling
Dussault et al. (2019)<br><br>Canada	Longitudinal study	To examine the discrepancy of self-reporting of age first gambled by asking same question four times across a year	Older youth had greater discrepancies, along with time passed. The authors caution researchers when using self-report to examine age first gambled among youth.	**Knowledge gap [more research in general]**<br>More research into how psychological-related variables could affect the discrepancies found in the age of initiation of gambling activity
Dussault et al. (2020)<br><br>Canada	Cross-sectional research	To examine the characteristics of youth poker players	Results found 4 classes/groups of players: 1 played only simulated poker (i.e. not for real money), 2 only at school, 3 only at home, 4 a diverse pattern. The authors emphasise that adolescent poker players should not be considered a homogenous group.	**Knowledge gaps [Sub-groups]**<br>More research on sub-groups of gambling (different forms of gambling)
Elton-Marshall et al. (2016)<br><br>Canada	Cross-sectional research	To examine online and land-based gambling behaviour among adolescents in 3 Canadian provinces prior to the implementation of legalised online gambling	Adolescents were engaging in online gambling at a significantly higher rate than has been previously found and were using technology to engage in simulated gambling.	**Knowledge gap [Research on specific factors: Gambling intentions/behaviours/actions]**<br>Further research is needed to understand whether adolescents are betting on the outcomes of video games or engaging in video games that include gambling for money, or both.<br>Further research is needed to identify which websites youth are accessing.
Estevez et al. (2020a)<br><br>Spain	Cross-sectional research	To investigate the severity of gambling and gambling related cognitions as predictors of emotional regulation and coping strategies in adolescents	Problem gamblers scored higher on gambling severity, emotional dysregulation, cognitive biases, and maladaptive coping strategies than non-problem gamblers.	**Knowledge gap [sub-groups]**<br>More research needed on female adolescent and young adult gambling involvement
Estevez et al. (2019)<br><br>Spain	Cross-sectional research	To investigate attachment and behavioural addictions in adolescents	Results showed parent-child attachment was negatively associated with all behavioural addictions: gambling, internet, video gaming.	**Knowledge gap [more research in general and intervention programs]**<br>Further research on the role of parental and peer attachment and its relationship to coping mechanisms. Development and evaluation of gambling intervention programs for children and adolescents
Estevez et al. (2020b)<br><br>Spain	Cross-sectional research	To examine the role of alexithymia in adolescents and young people and its relationship with gambling and attachment	Problem gamblers scored higher on substance, alcohol, gaming disorders and alexithymia, and lower in parental attachment than non-problem gamblers. Alexithymia was found to mediate the negative relationship between parental	**Knowledge gap [more research in general and intervention programs]**<br>More research of parental attachment and problem gambling among adolescents, and development of targeted interventions

			attachment and problem gambling.	
Farhat et al. (2020a)<br><br>USA	Cross-sectional research	To examine gambling attitudes, health correlates, and excitement seeking among adolescents	Excitement-seeking gambling was associated with greater at risk/problem gambling, greater alcohol use, and more permissive giving cognitions than non-excitement seeking gambling.	**Knowledge gap [more research in general]**<br>More research needed on reasons why adolescents gamble
Farhat et al. (2020b)<br><br>USA	Cross-sectional research	To investigate self-injurious behaviour and gambling-related attitudes, perceptions, and behaviours among adolescents	Adolescents acknowledging self-harming behaviours were more likely to report at risk or problem gambling and more permissive giving gambling cognitions.	**Knowledge gap [Intervention programs]**<br>Research needed on development and evaluation of intervention programs for adolescent problem gambling targeting distress tolerance
Ferrara et al. (2019)<br><br>Italy	Commentary article	Commentary on risk of Gambling Disorders among adolescents	Gambling has become a significant public health issue and over the past 25 years the phenomenon of gambling among children and adolescents has grown, however, the issue remains hidden in too many societies and among health care professionals.	**Knowledge gap [more research in general]**<br>Research involving collaboration between scientific societies, governments and stakeholders, and use research findings to design effective social policies and public health intervention options
Frisone et al. (2020)<br><br>Italy	Literature review	Gambling in adolescence a narrative review of the last 20 years	Males more likely to have at risk or problem gambling. Review examines association of gender, drug use, technology, age differences, impulsivity/sensation seeking, role of society as a risk factor.	**Knowledge gap [Intervention programs]**<br>Research needed on development and evaluation of intervention programs
Gavriel-Fried et al. (2015)<br><br>Israel	Cross-sectional research	To examine the link between competitive sports and gambling behaviours	Significant association found between involvement in competitive sports and gambling frequency for both genders. Link with problem gambling only for males.	**Knowledge gaps [Intervention programs]**<br>A need for development and evaluation of the integration of gambling prevention programs among youths who are involved in competitive sports
Ginley et al. (2015)<br><br>USA	Cross-sectional research	To explore college students' beliefs about gambling	Males more likely to have gambled past year than females and report higher frequency of gambling. Adverse consequences from gambling reported by 15% of males compared to only 4% of females.	**Knowledge gap [More research in general]**<br>Further research on young peoples' gambling expectancies and association with risky gambling. Also, longitudinal research.
Giralt et al. (2018)<br><br>Germany	Cross-sectional research	To examine the prevalence of problem gambling among German students and use of different forms of gambling	Past 12-month participation in gambling was 40% with PG rate of 1.7 and 2.2 in the two States surveyed. Males with background of migration more at risk and displayed greater psychopathological symptoms. Slots and online associated with more problem gambling.	**Knowledge gap [Longitudinal]**<br>Need for longitudinal research to examine believes about gambling over time
Gonzalez-Roz et al. (2017)<br><br>Spain	Cross-sectional research	To examine gambling prevalence and behaviour among Spanish adolescents	Having a relative with gambling problems predicted risk gambling. Living with only one parent or no parents and participation with EGMs during past year predicted problem gambling. Mixed-mode gambling predicted both at risk and	**Knowledge gap [sub-groups]**<br>Further research on sub-groups of gamblers re how they access gambling e.g. land-based vs online only vs mixed modes of access. Also, intervention programs.

			problem gambling.	
Gonzalez-Sicilia et al. (2020)<br><br>Canada	Cross-sectional research	Investigation of association between parental smoking and child gambling	Early childhood smoke exposure corresponded to 18% increased chances of gambling participation at age 12.	**Knowledge gap [more research in general]**<br>More research on association between two major public health issues: smoking and problem gambling. Design and evaluation of relevant public health campaigns
Gori et al. (2015)<br><br>Italy	Cross-sectional research	To examine the relationship between adolescent gambling severity and attitudes	Frequent exposure to media messages may result in normalisation of gambling. At-risk gamblers more likely to be involved in heavy drinking and use of sedatives. 53% engaged in gambling during the past year.	**Knowledge gap [Intervention programs, public health]**<br>Development and evaluation of public health campaigns targeting youth gambling
Hanss et al. (2015)<br><br>Norway	Cross-sectional research	To investigate the prevalence and correlates of PG, including motivational variables (future gambling intentions, attitudes toward gambling, and gambling-related knowledge) among 17-year-old Norwegians	26% had gambled during the previous month. 20.3% gambled with no problems, 4.1% at-risk and 1.5 moderate risk and 0.2% problem gambling. Males more likely to be at-risk or problem gamblers. Prevalence.<br>Motivational variables (future gambling intentions, attitudes toward gambling, and gambling-related knowledge) distinguished best between those who did not gamble, non-problem gamblers, and risk and problem gamblers.	**Knowledge gap [longitudinal]**<br>Longitudinal research needed to examine attitudes over time, and alternative measures of gambling behaviour could be used other than self-report (e.g. parental ratings)
Ho (2017)<br><br>Ethiopia	Cross-sectional research	To examine the risk factors of adolescent pathological gambling	Social poker and gambling in Amusement Gaming Centres on gambling type machines were predictive of problem gambling among adolescents. Internet gambling was not predictive of problem gambling.	**Knowledge gap [intervention programs, public health]**<br>Development and evaluation of responsible gambling practices from public health perspective to prevent underage gambling are needed.
Huic, A., et al. (2017)<br><br>Croatia	Cross-sectional research	To investigate gambling behaviour among adolescent girls in Croatia and examine psychosocial predictors	7.4% regular gamblers (once a week or more), 11.2% mild problems, 3.2% serious problems. 63% had ever gambled in their life (once a year or less and once a month or less combined), scratchies and lotto most preferred among lifetime gamblers with sports betting more preferred among regular gamblers.	**Knowledge gap [intervention programs]**<br>More research needed on needs of female adolescent gamblers to guide development of specific intervention programs, and longitudinal research is needed.
Jaisoorya et al. (2017)<br><br>India	Cross-sectional research	To examine gambling behaviour among high school students in India	27.9% of students had ever gambled, 7.1% problem gamblers. Of those who had ever gambled, 25.2% were problem gamblers (one in four). Problem gamblers were more likely to be males, have academic failure, higher rates of alcohol and tobacco use, and psychological distress including suicidality.	**Knowledge gap [more research in general]**<br>More research needed on adolescent problem gambling in India given the reported prevalence
Jauregui &	Cross-sectional	To examine the role of	Difficulties in emotional regulation mediated the	**Knowledge gap [intervention programs]**

Estevez (2020)<br><br>Spain	research	attachment, coping, and emotion Regulation in gambling among adolescents and young people	relationship between gambling motives and gambling severity. Attachment with parents and peers was negatively associated with problem gambling. Emotional regulation difficulties were positively related to problem gambling.	More research needed on development and evaluation of intervention programs
Jauregui et al. (2020)<br><br>Spain	Cross-sectional research	To investigate gambling motives and their association with addictive disorders and negative and positive mood in youth	Higher scores on problem gambling were associated with higher scores in enhancement (seeking to increase positive emotion), coping and social motives.	**Knowledge gap [intervention programs]**<br>More research needed on development and evaluation of intervention programs
Kaltenegger et al. (2019)<br><br>Sweden	Cross-sectional research	To investigate the relationship between impulsivity and risky gambling	Impulsivity was a risk factor for at risk gambling and episodic drinking.	**Knowledge gap [intervention programs]**<br>Prevention measures should address adolescents who exhibit risk factors for gambling and aim at reducing psychological problems**.**
Kang et al. (2019)<br><br>Korea	Cross-sectional research	To examine gambling factors related with the level of adolescent problem gamblers	Risk factors were nearby gambling facilities, personal relationship with people that gamble, being male more likely to have a problem with gambling.	**Knowledge gap [intervention programs]**<br>Research needed on development of interventions which target not only individuals, but environmental strategies e.g. accessibility and regulation – more research on the environmental factors
Kang et al. 2020)<br><br>Korea	Cross-sectional	Examination of gambling subgroups among Korean out-of-school adolescents	Three subgroups emerged: rarely gambled, immediate gain gambling, broad gambling. Immediate gain gambling scored higher on problem gambling severity and time and money spent gambling. Broad gamblers gambled mostly for fun and excitement, not to win money. Immediate gain gamblers gambled mainly to win money.	**Knowledge gap [sub-groups vulnerable adolescents]**<br>More research needed on vulnerable sub-groups of adolescent gamblers: e.g. those not attending formal education who are generally not picked up in school/educational institution surveys
Kam et al. (2017)<br><br>China	Cross-sectional research	To investigate gambling behaviour among college and university students in China	Most frequently reported gambling activities were mahjong (61.8%), soccer matches (40.2%) and Mark Six lottery (37.2%. Males were more likely to report gambling problems. Most problem gamblers had their first bet before age 14 years.	**Knowledge gap [Intervention programs]**<br>Development and evaluation of preventative programs on university campuses
Kim et al. (2016)<br><br>Canada	Qualitative research	To explore the potential link between social casino games and online gambling	While many young adults felt immune to effects of social casino games, there was a general consensus that social casino games might facilitate the transition to online gambling among younger teenagers (aged 12-14), due to the ease of accessibility and early exposure.	**Knowledge gap [More research in general]**<br>More studies are needed to further examine the link between social casino games and online gambling
King & Delfabbro (2016)	Cross-sectional research	To examine the types and extent of perceived parental	Financial gambling was usually facilitated by a parent, particularly for scratch tickets and	**Method gap [Qualitative research; Experimental research]**<br>**Knowledge gap [Sub-populations]**

Australia		influences on adolescents' gambling activities, including whether there might exist differences across commercial and simulated types of activities	sports betting. Simulated gambling activities, such as social casino games, tended to be reported as being unsupervised by parents. Young people's perceptions of parents' measures to limit, restrict or oversee online and electronic activities were not significantly associated with youth simulated gambling. Their perceptions of parental influences were not significant predictors of problem gambling behaviours.	Clinical and qualitative studies of adolescents engaged in gambling, including populations from mental health settings, is needed in order to cross-validate survey data on adolescent problem gambling
Kristiansen & Trajberg (2017)<br><br>Denmark	Qualitative research	To explore how young people experience and respond to changes in gambling opportunities	Four overarching themes were identified: legislation, conditions of entry, proximity and technological innovations.	**Method gap [Qualitative research]**<br>Further research is needed:<br>To shed light on the dimensions of the online interaction between adolescents and gambling operators<br>To assess whether money-free online gambling encourages positive attitudes towards commercial gambling, and the effects of online advertising on young people, including minors<br>To explore perceptions and implications of free bonuses
Kristiansen et al. (2018)<br><br>Denmark	Qualitative research (longitudinal)	To explore the types of simulated games and gambling platforms used by adolescents, adolescent's experiences, motivations and behaviours vis a`-vis simulated gambling and the potential interrelationships between simulated and monetary forms gambling	Enjoying social interactional effects appeared to be the main reasons young people engage in simulated gambling games. The study documented characteristics of both a catalyst pathway and a containment pathway emphasizing that for some young people simulated gambling may increase the likelihood of involvement in real money gambling while it may decrease it for others.	**Knowledge gap [Research on specific factors: Psychological]**<br>More research is needed to explore and clarify the psychological mechanisms at play in various forms of online simulated gambling, which may help understand how players are induced to continue playing or to transition from simulated gambling to monetary gambling forms.
Kryszajtys et al. (2018)<br><br>Canada	Literature review	To review the literature on the relationship between delinquent behaviour and adolescent gambling between 2000 – 2016	Nine studies met inclusion criteria. Problem gambling and delinquent behaviours have common risk factors	**Knowledge gap**<br>More research on the relationship between gambling and delinquent behaviour is needed; in particular, the development and evaluation of targeted intervention strategies. No qualitative studies met inclusion criteria.
Li et al. 2018<br><br>Australia	Experimental design	To examine implicit association and explicit intention regarding gambling and sport among underage youth	An implicit association found between gambling and sport for both males and females. Stronger association among sports that are generally associated with gambling (football, basketball rather than gymnastics or archery). Further, a stronger association found among sports where gambling logo was sports relevant (Bet365) than	**Knowledge gap**<br>More research is needed on the impact of gambling marketing on adolescents.

			irrelevant (X-lotto).	
Livazovic & Bojčić (2019)<br><br>Croatia	Cross-sectional research	To examine the psychological, social and financial consequences of problem gambling among adolescents	Findings showed 3-7% of adolescents regularly participate in serious gambling activities. Greatest risk, older male adolescents who struggled academically and have lower educated fathers and report low family satisfaction and attend vocational training facilities.	**Knowledge gap [more research in general]**<br>More research is needed to further examine if education improving knowledge misconceptions about gambling helps reduce risk of developing problem gambling among adolescents.
Marchica et al. (2017)<br><br>USA	Cross-sectional research	Examined the relationship between regular participation (more than once a month) in sport-relevant gambling activities among adolescents and those identified as being at-risk for a gambling problem	Regular involvement in sports betting, fantasy sports betting, and daily fantasy sports betting among adolescents was associated with a higher risk of gambling problems. Further, although males participate more frequently in these activities, females who participate have a stronger likelihood of being at-risk. Students aged 16–19 years old are at a higher risk for developing a gambling problem compared to younger adolescents when regularly engaging in sports-related gambling. Moreover, regularly participating in daily fantasy sports is the strongest predictor of at-risk gambling behaviour in 13 to 15-year-old students. A hierarchical logistic regression supports that controlling for gender and age, all forms of sport-relevant gambling activities are significant predictors of at-risk gambling.	**Method gap [More representative samples/other sampling gaps]**<br>Future studies should investigate the participation rates and impact of fantasy sports (both daily and league based) among adolescents on a larger scale in order to identify how this growing activity may affect problem gambling behaviours.
Martinez-Loredo et al. (2019)<br><br>Spain	Cross-sectional research	To investigate gender differences of substance use and gambling patterns among adolescents	Sensation seeking was associated with higher gambling problems in both males and females and with alcohol among females.	**Knowledge gap [sub-groups and intervention programs]**<br>More research needed on different groups of gamblers, and the development and evaluation of treatments to target them
McBride & Derevensky (2016)<br><br>Canada	Cross-sectional research	To examine commonalities between gambling behaviour and problem gambling among video game players and between video game playing and addicted playing among gamblers	Gamblers, relative to non-gamblers, were more likely to play video games. Video game players were more likely than nonplayers to gamble. Both social and problem gamblers had higher rates of video game playing than did non-gamblers, and addicted gamers had higher rates of gambling than did social and non-gamers.	**Knowledge gap [Research on specific factors: Gambling intentions/behaviours/actions; Psychological]**<br>It would be of interest to determine the extent to which individuals distinguish between gambling and video game playing with respect to skill in determining the outcome.<br>Further research into motivations for playing on the basis of gender would help to inform treatment strategies.<br><br>**Method gap [More representative samples/other sampling gaps]**<br>Research with young people who are not in school would provide valuable insight into the gambling behaviour of young adults in general.
Melendez-Torres	Cross-sectional	To examine the prevalence of	Males gambled more frequently than females,	**Knowledge gap [More research in general and longitudinal**

et al. (2020)<br><br>UK	survey	gambling behaviours and their associations with socioemotional harm among 11-16 year olds	more affluent families had higher level of adolescent gambling, lower connection to school associated with gambling and socioemotional harms. Form gambling fruit/slot machines, private bets with friends for money, cards with friends for money, and lotto. Gambling socioemotional harms positively correlated with age, and this interaction was more pronounced for males.	**research]**<br>More research into how adolescents view gambling and whether this predicts future gambling problems in adulthood
Nekich & Ohtsuka (2016)<br><br>Australia	Qualitative research	To explore the experiences of young Australian adults who gamble regularly at gambling machine venues	Overall, gambling was seen as positive social activity. The results highlighted the tension between individual agency, societal expectation, and familial influence.	**Method gap [intervention strategies]**<br>**Development of intervention strategies needed and should involve significant others, family, friendship groups**<br>The findings need to be confirmed using more recent data and by comparing with other market data.
Oksanen et al. (2019)<br><br>Finland	Cross-sectional	To investigate gambling activities and association with behavioural risk factors and protective factors 15-25 year olds	Competent online gambling (involving some level of perceived skill: poker, sports, online casino) a risk for problem gambling.	**Knowledge gap**<br>More research needed on new forms of online gambling, distinguishing between perceived skill-based and chance-based, and related policy development.
O'Loughlin & Blaszczynski (2018)<br><br>Australia	Randomised experimental design	To compare the influence of gambling advertisements posted on social media by gambling operators, on social media by peers, and gambling operators print media, on male youth gambling attitudes and intentions	Gambling operator adverts more influential than peer posted adverts, on both traditional print and social media, suggesting greater regulation for such industry adverts on social media	**Method gap [intervention strategies, public health]**<br>More research is needed on the influence of gambling advertising on youth, and development and evaluation of interventions from a public health perspective.
Paleologou et al. (2019)<br><br>Greece	Cross-sectional research	To investigate problem gambling and concurrent emotional and behavioural problems among adolescents	Hyperactivity/attentional problems were related to problem gambling. Also related were emotional problems, peer problems, conduct problems. Prosocial scores were negatively associated with problem gambling.	**Knowledge gap**<br>Further investigation needed for concurrent emotional/behavioural problems in adolescents with gambling problems
Pallesen et al. (2016)<br><br>Norway	Longitudinal study	To examine factors which influence changing attitudes towards gambling	Approval of gambling by close others at wave 1 was associated with more acceptant attitudes at wave 2, and males developed more acceptant attitudes. Participation of gambling at wave 1 associated with greater development of acceptant attitudes at wave 2.	**Knowledge gaps**<br>Males may develop more acceptant gambling attitudes than females in late adolescence therefore preventative strategies should take note of these findings. More longitudinal studies are needed to identify factors which can change gambling related attitudes over time
Passanisi et al. (2019)	Cross-sectional research	To investigate the relationship between	Results indicated emotional problems associated with problem gambling. Emotional problems,	**Knowledge gap**<br>More research to examine factors that explain adolescent gambling,

Greece		maladaptive personality traits and mindfulness deficits among adolescent regular gamblers	attentional problems, social dysfunction all significantly associated with gambling problems.	and more longitudinal research needed
Pisarka & Ostaszewski (2020)<br><br>Poland	Longitudinal study	To examine the factors associated with youth gambling	Most frequently reported were lotter, scratch cards, then sports betting. Males more likely to have risky gambling, other risk factors were sensation seeking and cyber bullying. Protective factors were positive relationship with parents and meaningful activities. Conclusion re practical implications was interventions which build positive relationships with parents and develop meaningful activities for adolescents. The rate of sports betting increased at time/wave 2, indicating that this type of gambling increases with age (over 2 years).	**Knowledge gap**<br>More research needed on adolescent gambling and associated sociodemographic factors
Raisamo et al. (2020)<br><br>Finland	Cross-sectional (comparative)	To examine changes in gambling expenditure and gambling-related harms among adolescents in Finland between 2011-2017	Significant reduction of gambling expenditure among 12-16 year olds. No change among 18 year olds. Among both boys and girls, most prevalent reported harm feeling ashamed, disrupt daily rhythm and disrupt school/work	**Knowledge gap**<br>Further regular updates are required due to the rapidly changing gambling landscape.
Rasanen et al. (2016)<br><br>Finland	Cross-sectional	To examine relationships between gambling frequency, health risk-taking and poor health, and support (parental, friends, school).	Poor health and health risk-taking associated with increased gambling. Social support from family, school related to decreased gambling boys and girls.	**Knowledge gap [treatment/intervention]**<br>Interventions are needed and should target other risk-taking into consideration, and increase social support from parents and schools.
Rash & McGrath (2017)<br><br>Canada	Cross-sectional research	To investigate the motives for not gambling among young adult non-gamblers	The results revealed eight specific motives for why participants chose not to gamble: 'financial reasons and risk aversion'; 'disinterest and other priorities'; 'personal and religious convictions'; 'addiction concerns'; 'influence of others' values'; 'awareness of the odds'; 'lack of access, opportunity, or skill'; and 'emotional distress'.	**Knowledge gap**<br>Need for design and evaluation of preventative strategies for gambling disorder
Reardon et al. (2019)<br><br>USA	Cross-sectional research	To examine personality factors associated with adolescent gambling	Findings indicated that sensation seeking had the greatest predictive ability for problem gambling. The importance of sensation seeking as a vulnerability among adolescents with respect to developing problem with gambling, is emphasised.	**Knowledge gap [sub-groups]**<br>Further research is needed for each age group, and longitudinal research spanning several developmental periods is needed
Rider et al. (2019)	Cross-sectional research	To examine gambling behaviours among	TGD-male assigned at birth had higher gambling participation and prevalence of problem gambling	**Knowledge gap [sub-groups/vulnerable groups]**<br>More research is needed for vulnerable sub-groups of adolescent

USA		transgender gender diverse (TGD) and cisgender adolescents	then female at birth assigned TGD adolescents. Both male and female assigned at birth TGD adolescents had higher problem gambling than cisgender males and females.	gamblers along with the development and evaluation of targeted intervention programs.
Sancartier et al. (2019)<br><br>Canada	Cross-sectional research	To examine gambling behaviour among young adults aged 19 to 20 years	Male problem gambler reported lack of social support as barrier to recovery, females reported relying more on escape and avoidance behaviours. Being male increased the odds of being a moderate/high risk problem gambler by 25 times. Higher alcohol use among high risk problem gambling males. Low risk gambling was associated with drug use but not high-risk gambling.	**Knowledge gap [sub-groups of gamblers and intervention programs]**<br>Intervention programs are needed to target different groups of gamblers: low risk and high risk. Also, more research is needed to investigate how gender and problem gambling severity interact.
Sarti & Triventi (2017)<br><br>Italy	Cross-sectional research	To examine the acceptability of gambling activities and compare social factors with psychological factors	Results suggest social factors more influential than psychological factors.	**Knowledge gap [More research in general]**<br>Further research needed concerning the influence of social norms which may influence young peoples' acceptance of gambling as a legitimate activity. Longitudinal research is needed.
Savolainen et al. (2019)<br><br>Finland	Cross-sectional research	To examine the role of perceived social support and peer group identification in problem gambling	Adolescents who identified more strongly with online peer groups were more likely to have gambling problems than those who identified with offline peers. Adolescents with offline peers has lower problem gambling. Perceived social supports moderated these effects.	**Knowledge gap**<br>Longitudinal research required. Further research is needed to examine the role of social support and the specific type of social support e.g. family vs friends, peers.
Sharman et al. (2019a)<br><br>UK	Cross-sectional research	To investigate psychosocial correlates in treatment seeking problem gamblers	Results indicated that the younger age of gambling participation, the more severe the gambling problem was. Those who began gambling early were also more likely to abuse drugs, commit crime or have histories of violent behaviour, less likely to report a positive family upbringing environment and more likely to have a parent with a gambling and or drug/alcohol problem.	**Knowledge gap [more research in general]**<br>More research is needed to examine the nature of these associations: poor family environment, gambling participation at an earlier age, and their association with problem gambling.
Sharman et al. (2019)<br><br>UK	Literature review	To review psychosocial factors in disordered gambling among vulnerable populations	Young people and adolescents were one of the seven identified vulnerable groups. Risk factors for young people comprised being male, parents that gamble, friends that gamble, lower family connectedness, being an only child, student employment, being an immigrant/foreign nationality, low grades at school, absence of food in house, major negative life events, history sexual abuse, child maltreatment and depression anxiety,	**Knowledge gap**<br>Research needed to examine transgenerational risk of problem gambling

			and involvement in competitive sports and video games with simulated gambling, exposure to gambling advertising.	
Sirola et al. (2019)<br><br>Finland	Cross-sectional research	To investigate loneliness and online gambling-community participation of young social media users	Results indicated that loneliness moderated the association between daily gambling online gambling community participation (e.g. a Facebook group) and excessive gambling.	**Knowledge gap**<br>Research is needed to understand emerging group processes within online social communities, and communities' significance in their users' everyday life, and to investigate how an increased understanding can be utilised in a harm-reductive manner.
Spangberg & Svensson (2020)<br><br>Finland	Cross-sectional	To compare gambling activity among 16-year-olds in Finland, Denmark, Iceland, Norway and Sweden	Cross-country differences found, Sweden lowest rate, parental monitoring a protective factor	**Knowledge gap**<br>More research is needed on social, economic and cultural factors associated with gambling involvement and problem gambling.
Tagoe et al. 2018<br><br>Africa	Qualitative research	To examine the understanding, initiation, and perceived benefits of gambling	Participants variously perceived betting on sports as a game that involves chance, money, and electronic gadgets. The perceived benefits of gambling included money, socialization, positive emotion, expression of knowledge, and love of football. Gamblers reported gambling helped to manage stress.	**Method gap [Intervention programs]**<br>Further research on the development and evaluation of programs which educate youth on adaptive coping strategies and the need for policies and programs to provide better alternatives to youth unemployment
Tara et al. (2016)<br><br>Canada	Cross-sectional research	To examine internet and land-based gambling among adolescents	The overall prevalence of online gambling among all adolescents was 9.4 %, the most common form was sports. Gambling online is common among adolescents despite restrictions.	**Knowledge gap [Intervention programs]**<br>Development and evaluation of intervention programs needed
Vitaro et al. (2019)<br><br>Canada	Longitudinal twin study	To examine gambling behaviour and substance use during late adolescence in a genetically informed twin study	Findings indicate that common genetic factors largely accounted for gambling participation and substance use at ages 17 and 19. Substance use and gambling participation share a common genetic component that account for their concurrent longitudinal links during late adolescence.	**Knowledge gap**<br>Future research is needed to examine what specific phenotypes may be associated with development of gambling participation and substance use.
Walters (2020)<br><br>USA	Cross-sectional research	To examine the relationships between parental gambling, child gambling and child delinquency	The association between child delinquency and gambling participation grew in proportion to the strength of parental participation in gambling activities.	**Knowledge gap [longitudinal research]**<br>Longitudinal research is needed to confirm these findings and establish causal direction of variables.
Wardel (2019)<br><br>UK	Literature review	To review qualitative research on youth gambling	Studies on perceptions highlighted the normalisation of gambling among youth and its embeddedness in everyday life. The review indicates young people's perceptions of gambling differ from legal and legislative definitions, which risks underestimating the nature and extent of	**Knowledge gap**<br>More research needed to increase our understanding of how technology shapes young peoples' perceptions and attitudes, and how, aside from accessibility, the how the techno-ecosystem leads to involvement and risky gambling

			youth gambling.	
Weinberger et al. (2017)<br><br>USA	Experimental design study	To explore gambling behaviours and attitudes among adolescents and relationship with smoking status	At risk problem gamblers were more likely to be smokers and report parental approval of gambling.	**Knowledge gap**<br>Further longitudinal research is needed to examine the influence of peer involvement on the occurrence of gambling and smoking among high school students.
Wu et al. (2019)<br><br>Hong Kong	Cross-sectional research	To examine social beliefs and their influence on adolescents' propensity to gamble	The results provide evidence for the importance of social beliefs in formulating specific gambling cognitions and gambling behaviours.	**Knowledge gap [intervention programs]**<br>The findings need to replicate with a larger representative sample. Findings can be used to inform treatment programs for adolescents.
Yu & Fu (2019)<br><br>Hong Kong	Cross-sectional research	To examine the relationships between disordered gambling, internet gaming addiction, and illusory control	Results suggested that non-gambling-related internet usage was not related to disordered gambling and using the internet for gambling related activities (gathering information – not gambling) was related to disordered gambling.	**Knowledge gap**<br>More research needed to examine the relationship between loot boxes and gambling
Yu & Ma (2019)<br><br>Hong Kong	Literature review	To review the literature on youth gambling in Hong Kong	Parental problem gambling is positively related to children's gambling and mental health (depression anxiety stress).	**Knowledge gap [vulnerable groups]**<br>Most youth studies use secondary school samples. More research is needed to examine 'hidden' youth who may be at greater risk.
Zhai et al. (2020)<br><br>USA	Cross-sectional research	To examine substance use, gambling, and violence among adolescents	Gambling participation past 12 months was associated with drug use and violence including carrying weapon and fighting for both males and females.	**Knowledge gap**<br>More research needs to examine link between delinquent behaviour and problem gambling.
Zhai et al. (2019)<br><br>USA	Cross-sectional research	To investigate the relationship between weapon carrying and problem gambling among high school students in USA	Weapon carriers had higher prevalence at risk problem gambling, greater parental approval of gambling, greater permissive gambling cognitions, and more family concerns compared to non-weapon carriers.	**Knowledge gap**<br>More research is needed to examine relationship between violence and gambling among adolescents. Development of targeted interventions are needed for at risk youth.

Supplementary File 3: Database Search Outputs

<u>Original Search</u>

DATABASE 1: MEDLINE

Search Strategy: Medline

#	Searches	Results
1	GAMBLING/	4899
2	((patholog* or problem* or addict* or compulsiv* or impulsive* or crav* or disorder*) adj7 gambl*).tw,kw.	4097
3	(ludomania or ludopath*).tw,kw.	3
4	(gambl* or betting or wagering or pokie* or lotter* or casino* or keno).tw,tw.	9907
5	(machine adj3 (gaming or fruit or slot)).tw,tw.	283
6	(video* and lotteri*).tw,kw.	8
7	1 or 2 or 3 or 4 or 5 or 6 or 7	11481
8	Video Games	1041
9	7 and 8	60
10	7 or 9	11481
11	(treat* or efficacy or effect* or "harm minimisation" or "responsible gambling" or attitude* or behavio?r* or prevalence or population or harm or policy or regulation).tw,kw.	11316517
12	BEHAVIOR THERAPY/	26395
13	Harm Reduction/	2499
14	Treatment Outcome/	866652
15	SELF EFFICACY/	17633
16	PSYCHIATRIC REHABILITATION/ or REHABILITATION/	17900
17	"PATIENT ACCEPTANCE OF HEALTH CARE"/	40306

18	Public Opinion/	17737
19	INTENTION/	9689
20	Therapeutics/	8323
21	TECHNOLOGY/ or INFORMATION TECHNOLOGY/	8802
22	((trend* or emerg* or future* or interactive or innovat*) and technolog*).tw,kw.	94461
23	PREVALENCE/ or Prevalence.tw,kw.	624596
24	Public Health/ or Public Health Administration/ or Public Health Practice/ or exp public policy/ or Community Health Services/ or Community health planning/ or Health Promotion/	297039
25	(public adj2 (health or policy or policies)).tw,kw.	212479
26	9 or 10 or 11 or 12 or 13 or 14 or 15 or 16 or 17 or 18 or 19 or 20 or 21 or 22 or 23	11918216
27	8 and 24	8194
28	limit 25 to (english language and humans and yr="2015 -Current")	1119

DATABASE 2: Emcare

Database(s): Ovid Emcare 1995 to 2018 week 40
Search Strategy:

#	Searches	Results
1	gambling/	1141
2	pathological gambling/	2790
3	((patholog* or problem* or addict* or compulsiv* or impulsive* or crav* or disorder*) adj7 gambl*).tw,kw.	2455
4	(ludomania or ludopath*).tw,kw.	0
5	(gambl* or betting or wagering or pokie* or lotter* or casino* or keno).tw,tw.	5571
6	(machine adj3 (gaming or fruit or slot)).tw,tw.	163
7	(video* and lotteri*).tw,kw.	6
8	or/1-7	5959

9	video game/	833
10	9 and 8	12
11	10 or 8	5959
12	(treat* or efficacy or effect* or "harm minimisation" or "responsible gambling" or attitude* or behavio?r* or prevalence or population or harm or policy or regulation).tw,kw.	2649891
13	behavior therapy/	14483
13	harm reduction/	3804
14	treatment outcome/	211803
15	self concept/	38547
16	psychosocial rehabilitation/ or rehabilitation/	49006
17	"PATIENT ACCEPTANCE OF HEALTH CARE"/	22715
18	public opinion/	4138
13	behavior/	24459
20	therapy/	134539
21	technology/	56811
22	information technology/	9465
23	((trend* or emerg* or future* or interactive or innovat*) and technolog*).tw,kw.	36419
24	PREVALENCE.tw,kw. or prevalence/	247143
25	public health/	81248
26	public health service/	24851
27	exp policy/	70928
28	community care/	21217
29	health care planning/	33055
30	health promotion/	42165
31	(public adj2 (health or policy or policies)).tw,kw.	101970

32	or/12-31	2965641
32	11 and 32	4545
33	limit 33 to (human and english language and yr="2015 -Current")	1119

DATABASE 3: PsycInfo

Search Strategy:

#	Searches	Results
1	gambling/ or pathological gambling/	7203
2	((patholog* or problem* or addict* or compulsiv* or impulsive* or crav* or disorder*) adj7 gambl*).ti,ab,id.	6012
3	(ludomania or ludopath*).ti,ab,id.	5
4	(gambl* or betting or wagering or pokie* or lotter* or casino* or keno).ti,ab,id.	12861
5	(machine adj3 (gaming or fruit or slot)).ti,ab,id.	433
6	(video* and lotteri*).ti,ab,id.	13
7	or/1-6	19411
8	computer games/	6574
9	7 and 8	193
10	7 or 9	13030
11	(treat* or efficacy or effect* or "harm minimisation" or "responsible gambling" or attitude* or behavio?r* or prevalence or population or harm or policy or regulation).ti,ab,id.	2552130
12	Behavior Therapy/	13407
13	Harm Reduction/	2914
14	Treatment Outcomes/	31374
15	Self-Efficacy/	20528
16	rehabilitation/	18612

17	Public Opinion/	8054
18	intention/ or behavioral intention/	14815
19	technology/ or information technology/	42661
20	((trend* or emerg* or future* or interactive or innovat*) and technolog*).ti,ab,id.	35497
21	PREVALENCE.ti,ab,id.	105331
22	Public Health/	19386
23	exp Government Policy Making/	41424
24	community services/ or community mental health services/ or public health services/	25984
25	health promotion/	22763
26	(public adj2 (health or policy or policies)).ti,ab,id.	56536
27	or/11-26	2631790
28	10 and 27	9985
29	limit 28 to (human and english language and yr="2015 -Current")	2260

DATABASE 4: Proquest: Health & Medicine databases

ab((((patholog* OR problem* OR addict* OR compulsiv* OR impulsive* OR crav* OR disorder*) NEAR/6 gambl*) OR ludomania OR ludopath* OR gambl* OR betting OR wagering OR pokie* OR lotter* OR casino* OR keno OR (machine NEAR/2 (gaming OR fruit OR slot)) OR (video* AND lotteri*)) AND ((treat* OR efficacy OR effect* OR "harm minimisation" OR "responsible gambling" OR attitude* OR behavio*r* OR prevalence OR population OR harm OR policy OR regulation OR ((trend* OR emerg* OR future* OR interactive OR innovat*) AND technolog*) OR (public NEAR/1 (health OR policy OR policies))))) AND (pd(20150101-20190201) AND PEER(yes)) or ti((((patholog* OR problem* OR addict* OR compulsiv* OR impulsive* OR crav* OR disorder*) NEAR/6 gambl*) OR ludomania OR ludopath* OR gambl* OR betting OR wagering OR pokie* OR lotter* OR casino* OR keno OR (machine NEAR/2 (gaming OR fruit OR slot)) OR (video* AND lotteri*)) AND ((treat* OR efficacy OR effect* OR "harm minimisation" OR "responsible gambling" OR attitude* OR behavio*r* OR prevalence OR population OR harm OR policy OR regulation OR ((trend* OR emerg* OR future* OR interactive OR innovat*) AND technolog*) OR (public NEAR/1 (health OR policy OR policies))))) AND (pd(20150101-20190201) AND PEER(yes))

n=1,388

DATABASE 5: Web of Science

# 9	4,317	(#4 OR #2 OR #1) *AND* **LANGUAGE:** (English)<br>*Indexes=SCI-EXPANDED, SSCI, A&HCI, CPCI-S, CPCI-SSH, ESCI, CCR-EXPANDED, IC Timespan=2015-2018*
# 8	7,289,985	#7 OR #6 OR #5<br>*Indexes=SCI-EXPANDED, SSCI, A&HCI, CPCI-S, CPCI-SSH, ESCI, CCR-EXPANDED, IC Timespan=1900-2018*
# 7	56,522	TI=((public NEAR/1 (health OR policy OR policies)))<br>*Indexes=SCI-EXPANDED, SSCI, A&HCI, CPCI-S, CPCI-SSH, ESCI, CCR-EXPANDED, IC Timespan=1900-2018*
# 6	26,268	TI=(((trend* OR emerg* OR future* OR interactive OR innovat*) AND technolog*))<br>*Indexes=SCI-EXPANDED, SSCI, A&HCI, CPCI-S, CPCI-SSH, ESCI, CCR-EXPANDED, IC Timespan=1900-2018*
# 5	7,227,506	TI=((treat* OR efficacy OR effect* OR "harm minimisation" OR "responsible gambling" OR attitude* OR behavio* OR prevalence OR population OR harm OR policy OR regulation))<br>*Indexes=SCI-EXPANDED, SSCI, A&HCI, CPCI-S, CPCI-SSH, ESCI, CCR-EXPANDED, IC Timespan=1900-2018*
# 4	3	TI=((video* AND lotteri*))<br>*Indexes=SCI-EXPANDED, SSCI, A&HCI, CPCI-S, CPCI-SSH, ESCI, CCR-EXPANDED, IC Timespan=1900-2018*
# 3	648	TI=((machine NEAR/2 (gaming OR fruit OR slot)))<br>*Indexes=SCI-EXPANDED, SSCI, A&HCI, CPCI-S, CPCI-SSH, ESCI, CCR-EXPANDED, IC Timespan=1900-2018*
# 2	18,223	TI=(ludomania OR ludopath* OR gambl* OR betting OR wagering OR pokie* OR lotter* OR casino* OR keno)<br>*Indexes=SCI-EXPANDED, SSCI, A&HCI, CPCI-S, CPCI-SSH, ESCI, CCR-EXPANDED, IC Timespan=1900-2018*
# 1	3,261	TI=(((patholog* OR problem* OR addict* OR compulsiv* OR impulsive* OR crav* OR disorder*) NEAR/6 gambl*))<br>*Indexes=SCI-EXPANDED, SSCI, A&HCI, CPCI-S, CPCI-SSH, ESCI, CCR-EXPANDED, IC Timespan=1900-2018*

n=975

DATABASE 6: Scopus

(((patholog* OR problem* OR addict* OR compulsiv* OR impulsive* OR crav* OR disorder*) NEAR/6 gambl*) OR ludomania OR ludopath* OR gambl* OR betting OR wagering OR pokie* OR lotter* OR casino* OR keno OR (machine NEAR/2 (gaming OR fruit OR slot)) OR (video* AND lotteri*)) AND (treat* OR efficacy OR effect* OR "harm minimisation" OR "responsible gambling" OR attitude* OR behavio* OR prevalence OR population OR harm OR policy OR regulation OR ((trend* OR emerg* OR future* OR interactive OR innovat*) AND technolog*) OR (public NEAR/1 (health OR policy OR policies)))

n=4,137

Updated Search Example Output (Repeated across databases)

DATABASE 1: MEDLINE

Search Strategy:

#	Searches	Results
1	Gambling/	5534
2	((patholog* or problem* or addict* or compulsiv* or impulsive* or crav* or disorder*) adj7 gambl*).tw,kw.	4769
3	(ludomania or ludopath*).tw,kw.	3
4	(gambl* or betting or wagering or pokie* or lotter* or casino* or keno).tw,tw.	11435
5	(machine adj3 (gaming or fruit or slot)).tw,tw.	318
6	(video* and lotteri*).tw,kw.	8
7	or/1-6	12099
8	Video Games/	5474
9	7 and 8	203
10	7 or 9	12099
11	(treat* or efficacy or effect* or "harm minimisation" or "responsible gambling" or attitude* or behavio?r* or prevalence or population or harm or policy or regulation).tw,kw.	12657514
12	behavior therapy/	27938
13	harm reduction/	3125
14	Treatment Outcome/	978524
15	self efficacy/	20388
16	psychiatric rehabilitation/ or rehabilitation/	18649
17	"patient acceptance of health care"/	47097
18	public opinion/	18643
19	intention/	11455
20	therapeutics/	8472
21	technology/ or information technology/	9961
22	((trend* or emerg* or future* or interactive or innovat*) and technolog*).tw,kw.	117855
23	Prevalence/ or prevalence.tw,kw.	716058
24	Public Health/ or Public Health Administration/ or Public Health Practice/ or exp public policy/ or Community Health Services/ or Community health planning/ or Health Promotion/	321137
25	(public adj2 (health or policy or policies)).tw,kw.	251642
26	or/11-25	13321568

27	10 and 26	8770
28	limit 27 to (english language and humans and yr="2019 -Current")	465
29	(elderly or old or aged or geriatrics).mp.	6136534
30	28 not 29	272

DATABASE 2: Emcare

Database(s): **Ovid Emcare** 1995 to 2020 Week 34
Search Strategy:

#	Searches	**286Results**
1	gambling/	1861
2	pathological gambling/	3161
3	((patholog* or problem* or addict* or compulsiv* or impulsive* or crav* or disorder*) adj7 gambl*).tw,kw.	3159
4	(ludomania or ludopath*).tw,kw.	1
5	(gambl* or betting or wagering or pokie* or lotter* or casino* or keno).tw,tw.	6892
6	(machine adj3 (gaming or fruit or slot)).tw,tw.	204
7	(video* and lotteri*).tw,kw.	6
8	video game/	1599
9	or/1-7	7355
10	8 and 9	43
11	10 or 9	7355
12	(treat* or efficacy or effect* or "harm minimisation" or "responsible gambling" or attitude* or behavio?r* or prevalence or population or harm or policy or regulation).tw,kw.	3175763
13	behavior therapy/	15352
14	harm reduction/	4938
15	treatment outcome/	230477
16	self concept/	44415
17	psychosocial rehabilitation/ or rehabilitation/	53418
18	"PATIENT ACCEPTANCE OF HEALTH CARE"/	25254
19	public opinion/	4536
20	behavior/	28051

21	therapy/	134293
22	technology/	28757429
23	information technology/	28810510
24	((trend* or emerg* or future* or interactive or innovat*) and technolog*).tw,kw.	28946134
25	PREVALENCE.tw,kw. or prevalence/	290300674
26	public health/	29197074
27	public health service/	29227797
28	exp policy/	293156873
29	community care/	23219
30	health care planning/	36714
31	health promotion/	47826
32	(public adj2 (health or policy or policies)).tw,kw.	129522
33	or/12-32	3552424
34	11 and 33	5736
35	limit 34 to (human and english language and yr="2019 -Current")	784
36	(elderly or old or aged or geriatrics).mp. [mp=title, abstract, heading word, drug trade name, original title, device manufacturer, drug manufacturer, device trade name, keyword]	1244435
37	35 not 36	650

DATABASE 3: PsycInfo

Database(s): **APA PsycInfo** 1806 to August Week 3 2020
Search Strategy:

#	Searches	Results
1	gambling/ or pathological gambling/	7951
2	((patholog* or problem* or addict* or compulsiv* or impulsive* or crav* or disorder*) adj7 gambl*).ti,ab,id.	6755
3	(ludomania or ludopath*).ti,ab,id.	5
4	(gambl* or betting or wagering or pokie* or lotter* or casino* or keno).ti,ab,id.	14252
5	(machine adj3 (gaming or fruit or slot)).ti,ab,id.	480
6	(video* and lotteri*).ti,ab,id.	13
7	computer games/	7569
8	or/1-6	14432
9	8 and 7	240
10	8 or 9	14432
11	(treat* or efficacy or effect* or "harm minimisation" or "responsible gambling" or attitude* or behavio?r* or prevalence or population or harm or policy or regulation).ti,ab,id.	2754253
12	Behavior Therapy/	14018
13	Harm Reduction/	3299
14	Treatment Outcomes/	34123
15	Self-Efficacy/	22966
16	rehabilitation/	20033
17	Public Opinion/	8598
18	intention/ or behavioral intention/	16844
19	technology/ or information technology/	46212
20	((trend* or emerg* or future* or interactive or innovat*) and technolog*).ti,ab,id.	40624
21	PREVALENCE.ti,ab,id.	117280
22	Public Health/	21637
23	exp Government Policy Making/	51188
24	community services/ or community mental health services/ or public health services/	27268
25	health promotion/	24840
26	(public adj2 (health or policy or policies)).ti,ab,id.	64174
27	or/11-26	2843944

28	10 and 27	11186
29	limit 28 to (human and english language and yr="2019 -Current")	594
30	(elderly or old or aged or geriatrics).mp. [mp=title, abstract, heading word, table of contents, key concepts, original title, tests & measures, mesh]	675372
31	29 not 30	549

DATABASE 4:

SH Psychosocial rehabilitation used for Psychiatric rehabilitation
SH not available Public Health Practice or Community health planning

#	Query	Limiters/Expanders	Results
S35	S33 NOT S34	Limiters - Published Date: 20190101-20201231; English Language<br>Expanders - Apply equivalent subjects<br>Search modes - Boolean/Phrase	510
S34	TX elderly OR old OR aged OR geriatrics	Limiters - Published Date: 20190101-20201231; English Language<br>Expanders - Apply equivalent subjects<br>Search modes - Boolean/Phrase	130,804
S33	S10 AND S31	Limiters - Published Date: 20190101-20201231; English Language<br>Expanders - Apply equivalent subjects<br>Search modes - Boolean/Phrase	687
S32	S10 AND S31	Expanders - Apply equivalent subjects	5,699

		Search modes - Boolean/Phrase	
S31	S9 OR S10 OR S11 OR S12 OR S13 OR S14 OR S15 OR S16 OR S17 OR S18 OR S19 OR S20 OR S21 OR S22 OR S23 OR S24 OR S25 OR S26 OR S27 OR S28 OR S29 OR S30	Expanders - Apply equivalent subjects<br>Search modes - Boolean/Phrase	2,736,385
S30	TI ((public N1 (health or policy or policies))) OR AB ((public N1 (health or policy or policies)))	Expanders - Apply equivalent subjects<br>Search modes - Boolean/Phrase	95,974
S29	(MH "Health Promotion")	Expanders - Apply equivalent subjects<br>Search modes - Boolean/Phrase	66,349
S28	(MH "Community Health Services")	Expanders - Apply equivalent subjects<br>Search modes - Boolean/Phrase	21,300
S27	(MH "Public Policy+")	Expanders - Apply equivalent subjects<br>Search modes - Boolean/Phrase	106,900
S26	(MH "Public Health Administration")	Expanders - Apply equivalent subjects<br>Search modes - Boolean/Phrase	5,157
S25	(MH "Public Health")	Expanders - Apply equivalent subjects<br>Search modes - Boolean/Phrase	48,676
S24	TI Prevalence OR AB Prevalence	Expanders - Apply equivalent subjects<br>Search modes - Boolean/Phrase	180,300

S23	(MH "Prevalence")	Expanders - Apply equivalent subjects<br>Search modes - Boolean/Phrase	92,957
S22	TI (((trend* or emerg* or future* or interactive or innovat*) and technolog*)) OR AB (((trend* or emerg* or future* or interactive or innovat*) and technolog*))	Expanders - Apply equivalent subjects<br>Search modes - Boolean/Phrase	29,803
S21	(MH "Information Technology")	Expanders - Apply equivalent subjects<br>Search modes - Boolean/Phrase	13,959
S20	(MH "Technology")	Expanders - Apply equivalent subjects<br>Search modes - Boolean/Phrase	17,857
S19	(MH "Therapeutics")	Expanders - Apply equivalent subjects<br>Search modes - Boolean/Phrase	4,016
S18	(MH "Intention")	Expanders - Apply equivalent subjects<br>Search modes - Boolean/Phrase	7,568
S17	(MH "Public Opinion")	Expanders - Apply equivalent subjects<br>Search modes - Boolean/Phrase	8,111
S16	(MH "Rehabilitation") OR (MH "Rehabilitation, Psychosocial")	Expanders - Apply equivalent subjects<br>Search modes - Boolean/Phrase	19,395
S15	(MH "Self-Efficacy")	Expanders - Apply equivalent subjects	21,237

		Search modes - Boolean/Phrase	
S14	(MH "Treatment Outcomes")	Expanders - Apply equivalent subjects<br>Search modes - Boolean/Phrase	346,840
S13	(MH "Harm Reduction")	Expanders - Apply equivalent subjects<br>Search modes - Boolean/Phrase	3,996
S12	(MH "Behavior Therapy")	Expanders - Apply equivalent subjects<br>Search modes - Boolean/Phrase	10,945
S11	TI (treat* or efficacy or effect* or "harm minimisation" or "responsible gambling" or attitude* or behavior* OR behaviour* or prevalence or population or harm or policy or regulation)) OR AB (treat* or efficacy or effect* or "harm minimisation" or "responsible gambling" or attitude* or behavior* OR behaviour* or prevalence or population or harm or policy or regulation))	Expanders - Apply equivalent subjects<br>Search modes - Boolean/Phrase	2,374,732
S10	S7 OR S9	Expanders - Apply equivalent subjects<br>Search modes - Boolean/Phrase	5,699
S9	S7 AND S8	Expanders - Apply equivalent subjects<br>Search modes - Boolean/Phrase	128
S8	(MH "Video Games")	Expanders - Apply equivalent subjects<br>Search modes - Boolean/Phrase	4,637
S7	S1 OR S2 OR S3 OR S4 OR S5 OR S6	Expanders - Apply equivalent subjects<br>Search modes - Boolean/Phrase	5,699

S6	TI ((video* AND lotteri*)) OR AB ((video* AND lotteri*))	Expanders - Apply equivalent subjects<br>Search modes - Boolean/Phrase	1
S5	TI ((machine N2 (gaming OR fruit OR slot))) OR AB ((machine N2 (gaming OR fruit OR slot)))	Expanders - Apply equivalent subjects<br>Search modes - Boolean/Phrase	212
S4	TI ((gambl* or betting or wagering or pokie* or lotter* or casino* or keno)) OR AB ((gambl* or betting or wagering or pokie* or lotter* or casino* or keno))	Expanders - Apply equivalent subjects<br>Search modes - Boolean/Phrase	5,228
S3	TI (ludomania OR ludopath*) OR AB (ludomania OR ludopath*)	Expanders - Apply equivalent subjects<br>Search modes - Boolean/Phrase	0
S2	TI (((patholog* or problem* or addict* or compulsiv* or impulsive* or crav* or disorder*) N6 gambl*)) OR AB (((patholog* or problem* or addict* or compulsiv* or impulsive* or crav* or disorder*) N6 gambl*))	Expanders - Apply equivalent subjects<br>Search modes - Boolean/Phrase	2,320
S1	(MH "Gambling")	Expanders - Apply equivalent subjects<br>Search modes - Boolean/Phrase	3,113

DATABASE 5: Proquest

Search Line one:
ab((((patholog* OR problem* OR addict* OR compulsiv* OR impulsive* OR crav* OR disorder*) NEAR/6 gambl*) OR ludomania OR ludopath* OR gambl* OR betting OR wagering OR pokie* OR lotter* OR casino* OR keno OR (machine NEAR/2 (gaming OR fruit OR slot)) OR (video* AND lotteri*)) AND ((treat* OR efficacy OR effect* OR "harm minimisation" OR "responsible gambling" OR attitude* OR behavio*r* OR prevalence OR population OR harm OR policy OR regulation OR ((trend* OR emerg* OR future* OR interactive OR innovat*) AND technolog*) OR (public NEAR/1 (health OR policy OR policies))))) AND (pd(20190101-20200201) AND PEER(yes)) or ti((((patholog* OR problem* OR addict* OR compulsiv* OR impulsive* OR crav* OR disorder*) NEAR/6 gambl*) OR ludomania OR ludopath* OR gambl* OR betting OR wagering OR pokie* OR lotter* OR casino* OR keno OR (machine NEAR/2 (gaming OR fruit

OR slot)) OR (video* AND lotteri*)) AND ((treat* OR efficacy OR effect* OR "harm minimisation" OR "responsible gambling" OR attitude* OR behavio*r* OR prevalence OR population OR harm OR policy OR regulation OR ((trend* OR emerg* OR future* OR interactive OR innovat*) AND technolog*) OR (public NEAR/1 (health OR policy OR policies))))) AND (pd(20190101-20200201) AND PEER(yes))

Search line 2
NOT (elderly OR old OR aged OR geriatrics)

DATABASE 6: Scopus

((TITLE-ABS-KEY ((((patholog* OR problem* OR addict* OR compulsiv* OR impulsive* OR crav* OR disorder*) W/6 gambl*) OR ludomania OR ludopath* OR gambl* OR betting OR wagering OR pokie OR lotter* OR casino* OR keno OR (machine W/2 (gaming OR fruit OR slot)) OR video* AND lotteri*))) AND (TITLE-ABS-KEY ((treat* OR efficacy OR effect* OR "harm minimisation" OR "responsible gambling" OR attitude* OR behavio*r OR prevalence OR population OR harm OR policy OR regulation OR ((trend* OR emerg* OR future* OR interactive OR innovat*) AND technolog*) OR (public W/1 (health OR policy OR policies)))))) AND NOT (ALL (elderly OR old OR aged OR geriatrics)) AND (LIMIT-TO (PUBYEAR , 2020) OR LIMIT-TO (PUBYEAR , 2019)) AND (LIMIT-TO (LANGUAGE , "English"))
